# Electrochemical sensing platform with gold nanoparticles capped by PDDA for benzyl alcohol determination

**DOI:** 10.1007/s00604-023-05690-6

**Published:** 2023-03-06

**Authors:** Lucía Abad-Gil, M. Jesús Gismera, M. Teresa Sevilla, Jesús R. Procopio

**Affiliations:** Departamento de Química Analítica y Análisis Instrumental, Facultad de Ciencias, Universidad Autónoma de Madrid. Avda. Francisco Tomás y Valiente, Avda. Francisco Tomás y Valiente, 7. E-28049, Madrid, Spain

**Keywords:** Gold nanoparticles, Response surface methodology, Central composite design, Electrochemical sensor, Linear sweep voltammetry, Benzyl alcohol

## Abstract

An electrochemical sensor has been developed, by modifying screen-printed carbon devices (SPCE) with photochemically synthesized gold nanoparticles (AuNP), to determine benzyl alcohol, a preservative widely used in the cosmetic industry. To obtain the AuNP with the best properties for electrochemical sensing applications, the photochemical synthesis was optimized using chemometric tools. A response surface methodology based on central composite design was used to optimize the synthesis conditions, as irradiation time, and the concentrations of metal precursor and the capping/reducing agent (poly(diallyldimethylammonium) chloride, PDDA). The anodic current of benzyl alcohol on SPCE modified with the AuNP was used as response of the system. The best electrochemical responses were obtained using the AuNP generated by irradiating for 18 min a 7.20 $\times$ 10^−4^ mol L^−1^ AuCl_4_^−^-1.7% PDDA solution. The AuNP were characterized by transmission electron microscopy, cyclic voltammetry and dynamic light scattering. The nanocomposite-based sensor formed by the optimal AuNP (AuNP@PDDA/SPCE) was used to determine benzyl alcohol by linear sweep voltammetry in 0.10 mol L^−1^ KOH. The anodic current at + 0.017 ± 0.003 V (vs. AgCl) was used as analytical signal. Detection limit obtained under these conditions was 2.8 µg mL^−1^. The AuNP@PDDA/SPCE was applied to determine benzyl alcohol  in cosmetic samples.

**Graphical Abstract:**

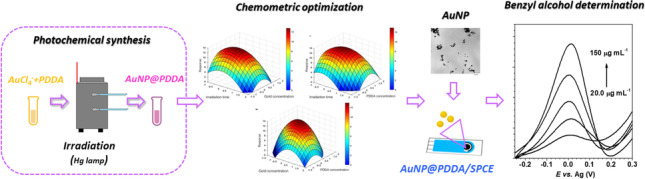

**Supplementary Information:**

The online version contains supplementary material available at 10.1007/s00604-023-05690-6.

## Introduction

Electrochemical sensors can be an interesting methodological option to determine organic or inorganic analytes in many different types of samples. The analytical performance of the sensors depends on the proper design of the sensing platform. Gold nanoparticles (AuNP) have gained interest as functional nanomaterial to develop electrochemical sensors due to their excellent electronic, magnetic, optic and catalytic properties [1, 2]. Nowadays, new green methods have been developed for the synthesis of AuNP diminishing the use of toxic reagents and the generation of hazard wastes. Photochemical synthesis has gained increasing attention due to its reproducibility, simplicity, mild synthesis conditions and lower reagents consumption [3, 4]. The most used radiation sources in photochemical synthesis are solar radiation and low-pressure mercury lamps [4–6]. The addition of some compounds with a great reducing activity due to the formation of radicals enhances the yield of the photochemical reaction and diminishes the irradiation time [7–9]. Different authors have demonstrated the influence of the size of the NP, or the capping agent in the electrochemical response of the AuNP based sensors [10–12]. Thus, synthesis conditions must be optimized to obtain AuNP with the best characteristics for an analytical purpose. Chemometric tools are widely used to optimize procedures reducing the number of experiments and consequently the consumption of reagents and costs. The response surface methodology (RSM) is the most relevant multivariate method for the optimization of analytical methodologies [13, 14].

This work presents the first AuNP-based electrochemical sensor for the determination of benzyl alcohol, a preservative widely added to cosmetics, but the maximum allowed concentration in these products is regulated since this compound is considered a chemical sensitizer [15, 16]. Benzyl alcohol is usually determined by HPLC methods coupled to diode-array (DAD) or mass spectrometer (MS) detectors [17, 18]. Electrochemical methods are an alternative to the chromatographic ones since they benefit from good properties such as low sample and reagents consumption, real-time analysis and good sensitivity in the measurements. These methods are also easy to implement and use. Several authors have studied the electrochemical response of benzyl alcohol on metal-based electrodes [19, 20]; however, there are no published electroanalytical methodologies for the determination of this preservative in real samples. AuNP used to modify the electrode were obtained by a photochemical synthesis route optimized by the RSM using the central composite design (CCD). The cationic polymer poly(diallyldimethylammonium) (PDDA) was added to the reaction media to enhance the yield of the reaction due to its reducing activity and to favour the homogeneous distribution of the nucleation sites, preventing the formation of agglomerates. The optimal AuNP were characterized by cyclic voltammetry, dynamic light scattering and transmission electron microscopy. An electrochemical sensor based on screen-printed carbon electrodes (SPCE) modified with AuNP was developed to determine benzyl alcohol by linear sweep voltammetry in cosmetic samples.

## Experimental section

### Chemicals and instrumentation

All reagents were analytical grade and used without further purification. Gold (III) chloride trihydrate (HAuCl_4_⋅3H_2_O), 20% aqueous PDDA chloride solution, benzyl alcohol, potassium hydroxide, phenoxyethanol, methylparaben, methylisothiazolinone, geraniol and linalool were from Sigma-Aldrich (Darmstadt, Germany). Salicylic, ascorbic and sulphuric acids were from Panreac (Barcelona, Spain). Aqueous stock solutions of AuCl_4_^−^ and benzyl alcohol were prepared at concentrations of 2.00 × 10^−2^ mol L^−1^ and 4000 µg mL^−1^, respectively. Millipore Milli-Q water (Type I with resistivity ≥ 18 MΩ cm) was used in this work.

A 705 UV Digester (Metrohm, Switzerland) equipped with quartz sample tubes, a high-pressure mercury lamp (500 W), an air–water cooling system and an irradiation time control unit was used for the photochemical synthesis of AuNP. The nanoparticles were characterized using the transmission electron microscopy (JEOL, JEM-1010, Japan) that operated at 100 kV and was equipped with digital cameras (4 K $\times$ 4 K), the Specord205 UV spectrophotometer (Analytik Jena, Germany) and the Zetasizer Ultra with Multi-Angle Dynamic Light Scattering capability (Malvern Panalytical, UK). The bipotentiostat/galvanostat μSTAT 400 (Metrohm, DropSens, Spain) equipped with the *DropView 8400* software was used to perform the electrochemical measurements. Disposable screen-printed carbon electrodes (SPCE, Metrohm, DropSens, DRP-110) formed by carbon as working electrode (4 mm diameter), Ag as pseudo-reference electrode and carbon as counter electrode were used as electrochemical platforms.

### Synthesis and characterization of AuNP

The optimal AuNP suspension was obtained by irradiating for 18 min, 2.0 mL of a 7.20 $\times$ 10^−4^ mol L^−1^ AuCl_4_^−^ solution in the presence of 1.7% PDDA. Surface plasmon resonance peak of AuNP was obtained by recording the UV–Vis spectra from 250 to 750 nm at 5.0 nm s^−1^, and 0.1 nm of Δλ. For transmission electron microscopy (TEM) measurements, a drop of the AuNP suspension was placed on a carbon-coated standard copper grid and dried at room temperature. The size of the nanoparticles was calculated using the *ImageJ* software and adjusting the data to a gaussian peak. Dynamic light scattering (DLS) measurements were performed using 12-mm square polystyrene cuvettes (DTS0012) and the dip cell (ZEN1002). A dilution 1:2500 with ultrapure water of the AuNP suspension was required for DLS measurements. AuNP were also electrochemically characterized using cyclic voltammetry between 0.0 V and + 1.75 V (vs. Ag) at 0.100 V s^−1^ in 0.50 mol L^−1^ H_2_SO_4_ by modifying the working electrode of SPCE with 4 µL of the suspension by a drop-casting method.

### Sensor preparation and application to benzyl alcohol determination

Prior the preparation of the sensor, SPCE were activated by recording 20 cyclic voltammograms from − 0.50 V to + 0.90 V (vs. Ag) at 0.500 V s^−1^ in 0.10 mol L^−1^ KCl and then rinsed with ultrapure water to remove any impurity and obtain a reproducible working electrode surface. After that, the working electrode surface was modified by drop-casting method with 4 μL of the AuNP suspension obtained after irradiating for 18 min a 7.20 $\times$ 10^−4^ mol L^−1^ AuCl_4_^−^ solution in the presence of 1.7% PDDA. Benzyl alcohol determination was performed on the AuNP-based sensor by linear sweep voltammetry (LSV) from − 0.25 V to + 0.30 V (vs. Ag) at 0.100 V s^−1^ in 0.10 mol L^−1^ KOH, using the anodic current at + 0.017 ± 0.003 V (vs. Ag) as analytical signal. Alkaline media was used as supporting electrolyte, since previous studies performed in our laboratory demonstrated that this compound is electroactive at alkaline pH values. A commercial micellar water and a facial tonic were analysed after simple dilution in the supporting electrolyte.

## Results and discussion

### Optimization of the photochemical synthesis of AuNP@PDDA

**Fig. 1:**
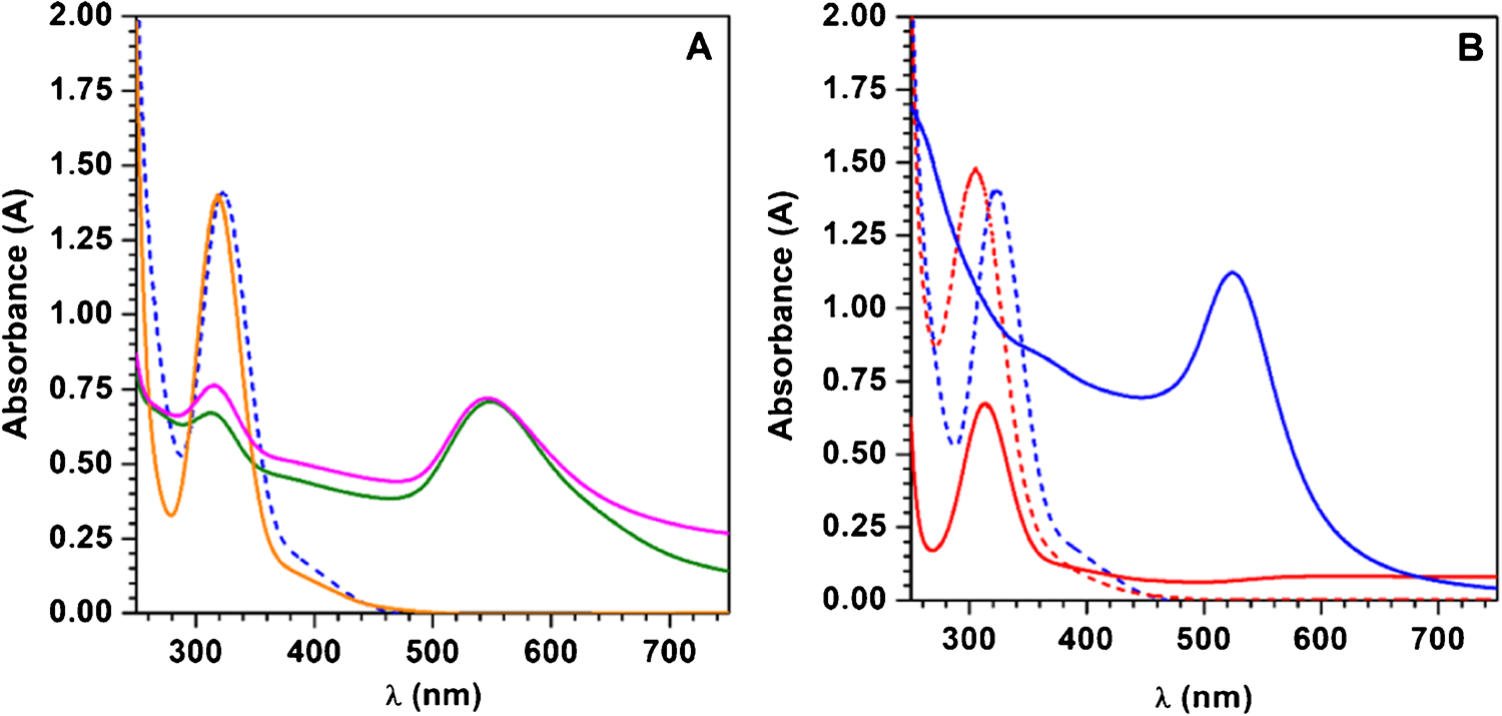
**A** Absorption spectra of a 5.00 $\times$ 10^−4^ mol L^−1^ AuCl_4_^−^ in 0.75% PDDA solution under different conditions:  non irradiated and at room temperature;  heated at 86 °C in the darkness; after irradiation for 1 min at  86 °C and  32 °C. **B** Absorption spectra of a 5.00 $\times$ 10^−4^ mol L^−1^ AuCl_4_^−^ aqueous solution  before and  after 15 min of irradiation and spectra of a 5.00 $\times$ 10^−4^ mol L^−1^ AuCl_4_^−^ 0.75% PDDA solution  before and  after 15 min of irradiation

Prior to photochemical synthesis optimization, the individual effects of temperature, irradiation and presence of PDDA on the synthesis of AuNP were evaluated. To evaluate the influence of the irradiation, a 5.00 $\times$ 10^−4^ mol L^−1^ AuCl_4_^−^ in 0.75% PDDA solution was heated for 15 min at 86 °C in the darkness. The effect of the temperature was investigated by irradiating, for 1 min, the Au (III)-PDDA solution at 32 °C and 86 °C. The influence of PDDA was studied by irradiating, for 15 min at 86 °C, a 5.00 $\times$ 10^−4^ mol L^−1^ AuCl_4_ solution in the presence and in the absence of the polymer. Fig. 1 shows the spectra of the Au (III) solution before and after the different assayed synthesis conditions. As can be seen, there are significant differences in the position of the band of the non-irradiated AuCl_4_^−^ aqueous solution (Fig. 1B red dashed line) and the Au (III)-PDDA solution (Fig. 1A, B blue dashed line). This displacement indicates the interaction between the AuCl_4_^−^ and the polymer. As can be seen in Fig. 1A, B, the surface plasmon resonance (SPR) peak due to the AuNP was only observed when the Au (III) solutions were irradiated in the presence of PDDA, and no significant differences were observed at the two temperatures assayed. These results confirm that the irradiation is required to obtain the AuNP, the temperature is not a critical point and PDDA favours the formation of AuNP acting both as capping and reducing agent. According to these results, the irradiation time and the concentrations of AuCl_4_^−^ and PDDA were selected as independent variables and optimized by RSM and CCD using the anodic current of a 200 µg mL^−1^ benzyl alcohol solution as the response of the system. (More information in Section S1 in Electronic Supplementary Material, ESM). The number of experiments required to optimize the three variables is significantly reduced from 125 tests needed with one-variable-at-a-time method to 18 tests required using RSM and CCD. According to the results obtained in the chemometric study, the maximum of the response was observed on the electrodes modified with the AuNP obtained in the centre point conditions, i.e. an irradiation time of 18 min, and concentrations of AuCl_4_^−^ and PDDA of 7.20 $\times$ 10^−4^ mol L^−1^ and 1.7%, respectively (Figure S2 in ESM). The variations observed in the response surfaces with the different values of the variables suggest that the synthesis procedure of the AuNP is a critical step to obtain nanoparticles with the best characteristics to develop electrochemical sensors for benzyl alcohol determination. In addition, the interactions between the irradiation time and the concentration of PDDA and between the concentration of the metal precursor and PDDA were significant, demonstrating the necessity of using chemometric tools to optimize the photochemical synthesis conditions.

### Characterization of the optimal AuNP@PDDA

**Fig. 2:**
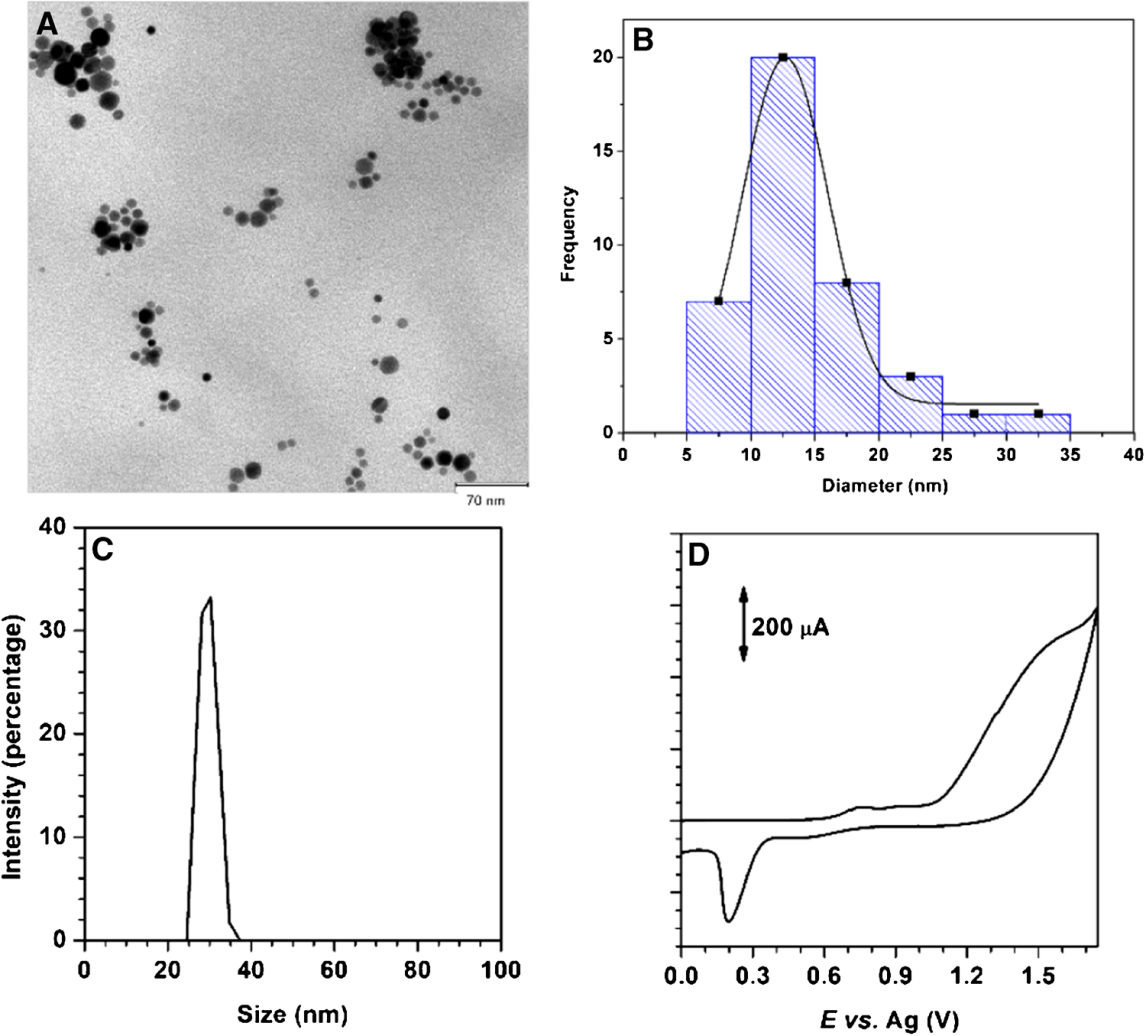
Characterization of the AuNP@PDDA suspension: (**A**) TEM image. (**B**) Size distribution from TEM image and gaussian fit (*n* = 40, in a surface area of 0.24 µm^2^). (**C**) Size distribution obtained from DLS measurements. (**D**) Cyclic voltammograms obtained in the AuNP@PDDA-based sensor in 0.50 mol L^−1^ H_2_SO_4_ at 0.100 V s^−^^1^

The AuNP suspension obtained in the optimal conditions (18 min of irradiation of a 7.20 $\times$ 10^−4^ mol L^−1^ AuCl_4_^−^-1.7% PDDA solution) was characterized by TEM, DLS and cyclic voltammetry. As can be seen in the TEM image and size distribution histogram (Fig. 2A, B) the AuNP present a narrow distribution and spherical shape. Some aggregates can be observed, but the mean size of the nanoparticles was 13 ± 2 nm (*n* = 40 in a surface area of 0.24 µm^2^). DLS measurements also showed a narrow size distribution of the AuNP (Fig. 2C). A higher AuNP size was obtained using DLS (32 ± 3 nm) than that calculated in TEM, due to DLS estimates the hydrodynamic radio of the particles, that depends on the size of the particles and other factors such as ionic strength of medium and surface structure of the particles. The zeta potential value was also estimated by DLS, obtaining a zeta potential value of + 24 ± 2 mV, so the AuNP suspension presented good stability and they were positively charged. After modification of SPCE with the AuNP suspension, the gold surface density (GSD) and the effective surface area (ESA) were calculated from the electrochemical characterization of AuNP following the procedure described in Sect. 2.2. using the area of the cathodic peak at + 0.21 ± 0.02 V (vs. Ag) (Fig. 2D). A mean value of GSD of 0.0056 ± 0.0004 µmol cm^−2^ was obtained. The ESA was determined as the ratio between the area of the cathodic peak at + 0.21 ± 0.02 V (vs. Ag) and the consumed charge for the desorption of oxygen (400 µC cm^−2^) [21]. The ESA value of the AuNP@PDDA-based sensor (0.45 ± 0.05 cm^2^) was higher than the geometric area of the unmodified SPCE (0.12 cm^2^).

The stability of the AuNP suspension and the modification of the AuNP@PDDA/SPCE platform was studied by recording the UV spectra at different times after irradiation and performing successive scans with AuNP@PDDA/SPCE in 0.50 mol L^−1^ H_2_SO_4_ at 0.100 V s^−1^, respectively. No significant differences in the position of SPR peak of AuNP were observed after 1 year, indicating the good stability of the obtained suspensions. Moreover, only a decrease of 10% in the cathodic peak area was observed after registering the successive scans for 1 h. This result demonstrates the adequacy of the procedure used to modify the working electrode surface since the loss of the nanoparticles was not significant. Thus, the drop-casting method used to prepare the AuNP@PDDA/SPCE sensor is easier and simpler to perform compared to more complex modification procedures such as covalent immobilization.

### Determination of benzyl alcohol with the AuNP@PDDA/SPCE sensors

**Fig. 3:**
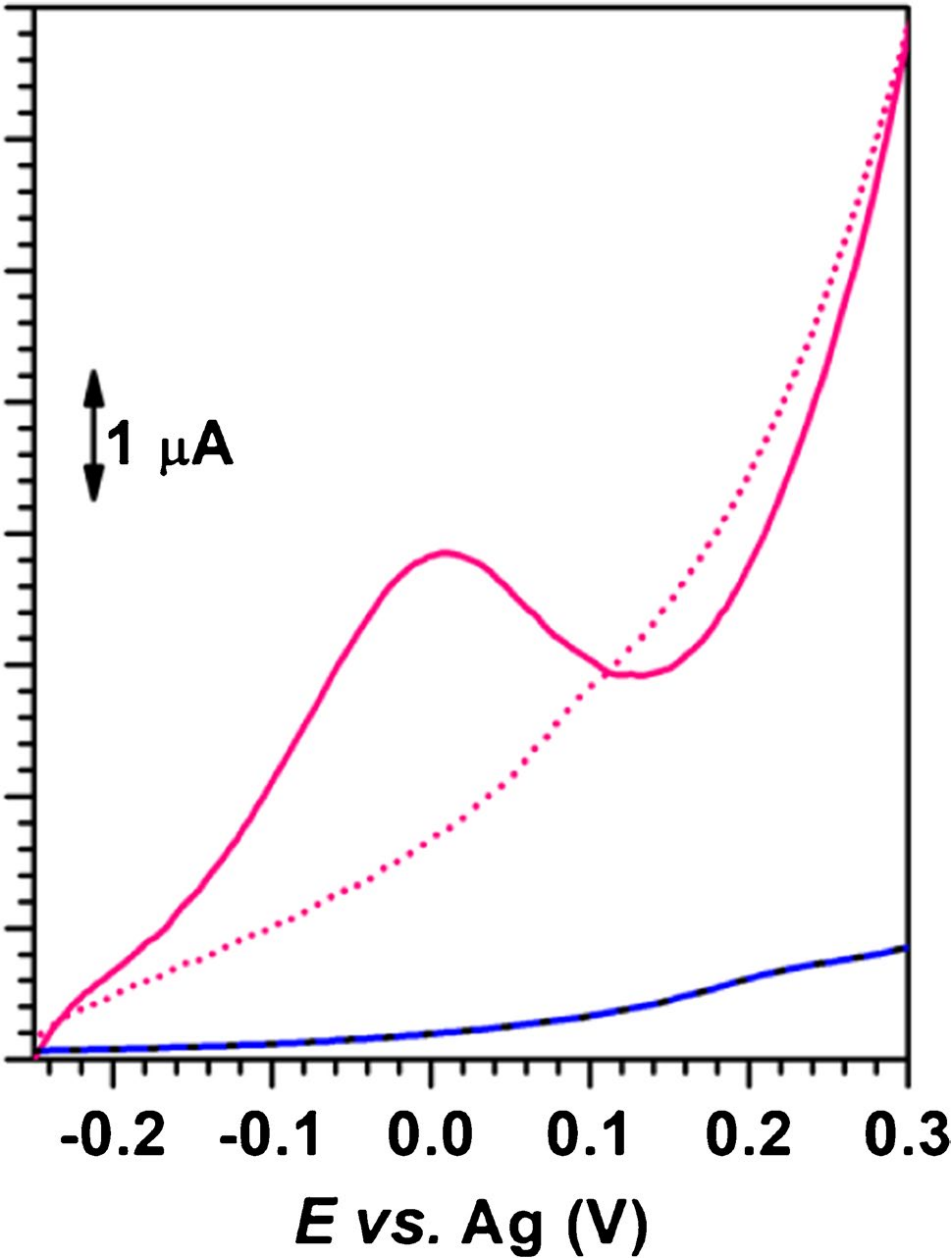
Linear sweep voltammograms of a 20.0 µg mL^−1^ benzyl alcohol in 0.10 mol L^−1^ KOH solution on SPCE (―), PDDA/SPCE  (overlapped signals) and AuNP@PDDA/SPCE  . Electrochemical response of AuNP@PDDA/SPCE in 0.10 mol L^−1^ KOH  . Scan rate: 0.100 V s^−^^1^

The electrochemical behaviour of a 20.0 µg mL^−1^ benzyl alcohol solution in 0.10 mol L^−1^ KOH was evaluated by LSV on a SPCE, a SPCE modified with PDDA (PDDA/SPCE) and a SPCE modified with the AuNP@PDDA suspension (AuNP@PDDA/SPCE). As can be seen in Fig. 3, the anodic peak at + 0.017 ± 0.003 V (vs. Ag) due to benzyl alcohol was only observed on the AuNP@PDDA-based sensor, confirming that this compound is not electroactive on carbon electrodes, but it is on gold electrodes.

**Fig. 4:**
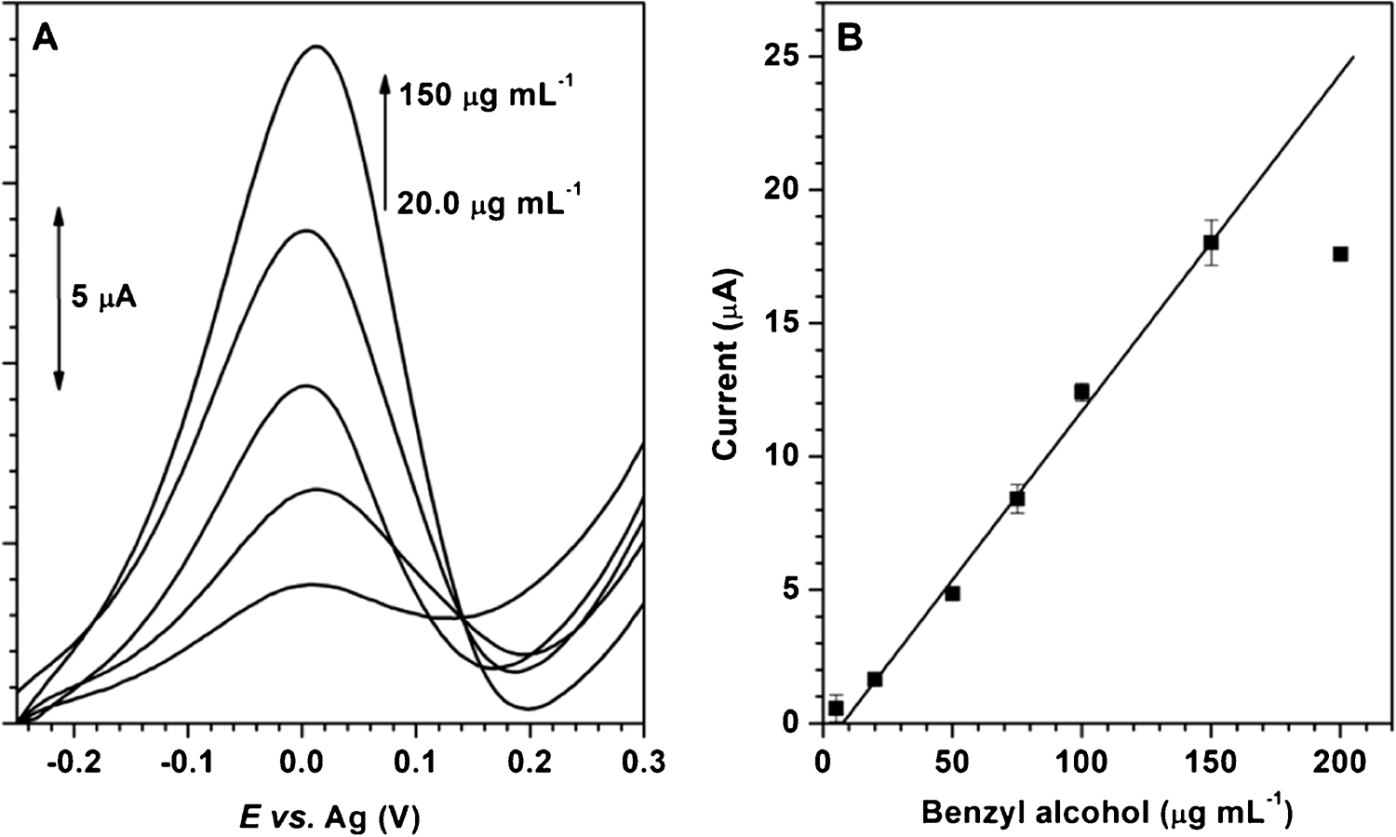
(**A**) Linear sweep voltammograms and (**B**) calibration plot (*n* = 3) of benzyl alcohol on AuNP@PDDA/SPCE. Supporting electrolyte: 0.10 mol L^−1^ KOH. Scan rate: 0.100 V s^−^^1^

Figure 4 presents the LS responses (voltammograms and calibration plot) of benzyl alcohol on AuNP@PDDA/SPCE at a concentration range between 5.00 and 200 µg mL^−1^. A good relationship (*r* = 0.9978) between the concentration of benzyl alcohol and the peak current up to a concentration of 154 µg mL^−1^ (upper limit of the linear range) was observed.

Table 1 shows the analytical parameters obtained for benzyl alcohol determination. The limits of detection (LOD) and quantification (LOQ) were calculated as the ratio between 3 and 10-times, respectively, the standard deviation of a benzyl alcohol solution of the lowest assayed concentration closed to LOD (5 µg mL^−1^) and the sensitivity of the method. The repeatability was calculated by successive measurements (*n* = 4) of 20.0 and 100.0 µg mL^−1^ benzyl alcohol solutions on the same sensor. To evaluate the reproducibility of the sensor, the LS voltammograms of 20.0 and 100.0 µg mL^−1^ benzyl alcohol solution were registered on four different AuNP@PDDA/SPCE platforms (*n* = 4). As expected, the repeatability and reproducibility are lower at concentration close to the LOQ.

**Table 1:** Analytical parameters for the determination of benzyl alcohol on AuNP@PDDA/SPCE by LSV

Sensitivity (µA mL µg^−1^)^a^	0.125 ± 0.005
ULLR^b^ (µg mL^−1^)	154
LOD (µg mL^−1^)	2.8
LOQ (µg mL^−1^)	9.3
Repeatability, RSD% (*n* = 4)	
at 20 µg L^−1^	7.0
at 100 µg L^−1^	2.8
Reproducibility, RSD% (*n* = 4)	
at 20 µg L^−1^	12
at 100 µg L^−1^	8.0

^a^Slope of the calibration plot ± standard deviation

^b^Upper limit of the linear range

As has already been indicated, there are not published electroanalytical methods for the determination of benzyl alcohol. For this reason, to evaluate the utility of the proposed sensor for benzyl alcohol determination, in Table 2 the analytical properties of the AuNP@PDDA/SPCE sensor-based method are compared with those reported for methods for benzyl alcohol determination in cosmetics. The linear intervals and repeatability are similar to those obtained by other methods. The here-obtained LOD was similar to [22–24] or even 10-times lower than that obtained by other authors using HPLC–DAD [25] or CE-UV [26]. Although, the LOD obtained using the proposed sensor was higher than those obtained in other HPLC methods [18, 27] it should be noted that electrochemical methods are simpler, faster and cheaper than those based on chromatography, and reagent consumption, specially of organic solvents, is much lower. The GC–MS methods present very low LOD values, as can be expected considering the characteristics of MS detector. On the other hand, according to the EU regulation [16], the LOD obtained using the AuNP@PDDA/SPCE is adequate to determine benzyl alcohol in cosmetic products with a lower dilution than that required in chromatographic measurements.

**Table 2:** An overview on reported methods for the determination of benzyl alcohol

Method	LOD, µg mL^−1^	Linear range, µg mL^−1^	%RSD	Reference
GC–MS	0.14^a^	Not indicated	< 10	[17]
HPLC–DAD	0.89	2.7–300	2.0	[18]
HPLC–DAD	20	75–454	0.67	[25]
UPLC-DAD	3.68	Not indicated	2.4	[22]
GC–MS	0.006	Not indicated	1.5	[28]
HPLC–DAD	2.0	8–48	3.3	[23]
UPLC-UV	4.38	15–100	2.16	[24]
HPLC- UV	0.556	5.0–175	0.483	[27]
UPLC-DAD	0.156	2.5–125	0.5	
CE-UV	20	10–200	3.7	[26]
AuNP@PDDA/SPCE-LSV	2.8	9.3–154	2.8	This work

^a^Estimated limit of detection

To evaluate the selectivity of the AuNP@PDDA/SPCE for the determination of benzyl alcohol some of the main usual ingredients in cosmetics (phenoxyethanol, methylparaben, linalool, geraniol, salicylic acid, ascorbic acid and methylisothiazolinone) were assayed as interfering substances. The LS voltammograms of a 50.0 µg mL^−1^ benzyl alcohol solution in 0.10 mol L^−1^ KOH were registered by triplicated in the presence of different concentrations of the interfering substances. The tolerance limit was defined as the maximum assayed concentration of the interfering substance at which no significant differences in the analytical signal, at 95%-confidence level, were observed, with respect to that obtained in the absence of the interfering substance. Table 3 shows the tolerance limits of the different assayed compounds expressed as the ratio between the concentration of the interfering compound to the concentration of BA. As can be seen, benzyl alcohol signal did not change in the presence of the maximum concentration of linalool assayed. The lowest tolerance limits were observed in the presence of methylisothiazolinone and ascorbic acid. The maximum allowed concentration of linalool, methylparaben, geraniol and methylisothiazolinone established in the EU Regulation No 1223/2009 on cosmetic products [16] is lower than the maximum allowed concentration of benzyl alcohol. According to the obtained tolerance limits, benzyl alcohol can be determined in cosmetics using the AuNP@PDDA/SPCE without interference of these compounds. For cosmetics that contain salicylic and/or ascorbic acid along with benzyl alcohol, a sample pretreatment will be required prior to the analytical measurement since the concentration of these substances can be higher than that of benzyl alcohol.

**Table 3:** Tolerance limits of foreign substances on AuNP@PDDA/SPCE sensor for 50.0 µg mL^−1^ benzyl alcohol

Interfering substance	Tolerance limit [substance]/[BA]
Linalool	10
Phenoxyethanol, methylparaben, salicylic acid, geraniol	1
Methylisothiazolinone, ascorbic acid	0.1

### Analysis of benzyl alcohol in cosmetic products

A micellar water and a facial tonic were analysed on AuNP@PDDA/SPCE by LSV in 0.10 mol L^−1^ KOH using the standard addition method. Prior the measurement, 1.0 mL of sample was diluted to 10.0 mL with the supporting electrolyte. The anodic peak observed at + 0.017 ± 0.003 V (vs. Ag) in the micellar water sample (Figure S3A in ESM) indicates the presence of benzyl alcohol in this sample, whereas this signal was not observed in the facial tonic. Both samples were fortified with 20.0 and 50.0 µg mL^−1^ of benzyl alcohol. For the micellar water, a significant difference between the slope values of the calibration plot using the standard addition method (slope of 0.152 ± 0.003 µA mL µg^−1^) and the external standard calibration method (0.125 ± 0.005 µA mL µg^−1^) was observed, indicating the presence of matrix effects. From the calibration plot obtained using the addition standard method (Figure S3B in ESM), the content of benzyl alcohol in the micellar water was calculated as 0.23 ± 0.02% (%wt./%wt.). This value does not exceed the limit established in the EU Regulation for benzyl alcohol in cosmetics (< 1.0%) [16]. For validation purposes, the micellar water was also analysed by HPLC–DAD obtaining a concentration of benzyl alcohol of 0.24 ± 0.02%. Thus, there are no significant differences (*p* = 0.05) between benzyl alcohol concentrations calculated using the AuNP@PDDA/SPCE sensor and the HPLC–DAD method.

To study the capability of sensor for BA determination in other matrices, the recoveries values in the facial tonic fortified at the two concentration levels of benzyl alcohol (20.0 and 50.0 µg mL^−1^) were evaluated. Recovery values of 86 ± 2% (at 50.0 µg mL^−1^) and 97 ± 5% (at 20.0 µg mL^−1^) were obtained. These results validate the accuracy and precision of the method using the AuNP@PDDA/SPCE sensor.

## Conclusions

A new electrochemical sensor based on the nanocomposite formed by PDDA capped AuNP photochemically synthesized has been developed for the determination of benzyl alcohol. AuNP with good and controlled characteristics for electrochemical sensing of benzyl alcohol were obtained by photochemical synthesis, a greener alternative method than the traditional ones widely used to synthesize these nanoparticles. The use of chemometric tools is required to optimize the experimental conditions for the photochemical synthesis of AuNP since interactions between these variables take place. In addition, a reduction of the costs is observed using the RSM with CCD since 18 tests were needed instead of 125 test that would have been required with the traditional methods for the optimization of 3 factors at 5 different levels. It is noteworthy that this is the first electrochemical sensor proposed for the determination of benzyl alcohol and the obtained LOD value is adequate to determine this preservative in cosmetics, considering the concentrations of this preservative established in the EU Regulation. According to the selectivity study, other components present in cosmetic formulations such as phenoxyethanol, can interfere in benzyl alcohol determination. This drawback can be overcome including a sample treatment procedure before the electrochemical measurement. The proposed AuNP@PDDA/SPCE was successfully applied to determine benzyl alcohol in a commercial micellar water with results statistically comparable to that obtained using HPLC–DAD methods. In addition, recovery values close to 100% were obtained when a commercial facial tonic was analysed. Thus, the AuNP@PDDA-based sensor can be considered a promising tool for sensing applications with a reduction of the cost compared to commercial AuNP-based screen-printed electrodes.

## Supplementary Information

Below is the link to the electronic supplementary material.

Supplementary file1 (DOCX 461 KB)

## Data Availability

The main data of the work is included in the manuscript and in the supplementary information. The rest of the information is confidential and is not available.

## References

[1] Alim S, Vejayan J, Yusoff MM, Kafi AKM (2018). Recent uses of carbon nanotubes & gold nanoparticles in electrochemistry with application in biosensing: a review. Biosens Bioelectron.

[2] Guo S, Wang E (2007). Synthesis and electrochemical applications of gold nanoparticles. Anal Chim Acta.

[3] Qiao J, Qi L (2021). Recent progress in plant-gold nanoparticles fabrication methods and bio-applications. Talanta.

[4] Jara N, Milán NS, Rahman A, Mouheb L, Boffito DC, Jeffryes C, Dahoumane SA (2021). Photochemical synthesis of gold and silver nanoparticles—a review. Molecules.

[5] Annadhasan M, Kasthurib J, Rajendiran N (2015). Green synthesis of gold nanoparticles under sunlight irradiation and their colorimetric detection of Ni^2+^ and Co^2+^ ions. RSC Adv.

[6] Huang WC, Chen YC (2008). Photochemical synthesis of polygonal gold nanoparticles. J Nanopart Res.

[7] Marin ML, McGilvray KL, Scaiano JC (2008). Photochemical strategies for the synthesis of gold nanoparticles from Au(III) and Au(I) using photoinduced free radical generation. J Am Chem Soc.

[8] Isaeva EI, Kiryukhina SN, Gorbunova VV (2013). Photochemical synthesis of silver and gold nanoparticles in polyhydric alcohols. Russ J Gen Chem.

[9] Eustis S, Hsu HY, El-Sayed MA (2005). Gold nanoparticle formation from photochemical reduction of Au^3+^ by continuous excitation in colloidal solutions: a proposed molecular mechanism. J Phys Chem B.

[10] Sierra-Rosales P, Torres R, Sepúlveda C, Kogan MJ, Squella JA (2018). Electrochemical characterization and electrocatalytic application of gold nanoparticles synthesized with different stabilizing agents. Electroanalysis.

[11] Escosura-Muñiz A, Parolo C, Maran F, Mekoçi A (2011). Size-dependent direct electrochemical detection of gold nanoparticles: application in magnetoimmunoassays. Nanoscale.

[12] Bonanni A, Pumera M, Miyahara Y (2011). Influence of gold nanoparticle size (2–50 nm) upon its electrochemical behavior: an electrochemical impedance spectroscopic and voltammetric study. Phys Chem Chem Phys.

[13] Tarley CRT, Silveira G, Santos WNL, Matos GD, da Silva EGP, Bezerra MA, Miró M, Ferreira SLC (2009). Chemometric tools in electroanalytical chemistry: methods for optimization based on factorial design and response surface methodology. Microchem J.

[14] Bezerra MA, Santelli RE, Oliveira EP, Villar LS, Escaleira LA (2008). Response surface methodology (RSM) as a tool for optimization in analytical chemistry. Talanta.

[15] Atwater AR, Petty AJ, Liu B, Green CL, Silverberg JI, DeKoven JG, Belsito DV, Reeder MJ, Sasseville D, Taylor JS, Maibach HI, Zirwas MJ, Marks JG, Zug KA, Fowler JF, Pratt MD, DeLeo VA, Warshaw EM (2021). Contact dermatitis associated with preservatives: retrospective analysis of north American contact dermatitis group data, 1994 through 2016. J Am Acad Dermatol.

[16] Regulation (EC) No 1223/2009 of the European parliament and of the council of 30 November 2009 on cosmetic products. http://data.europa.eu/eli/reg/2009/1223/2020-12-03

[17] Fardin-Kia AR, Zhou W (2020). Development and validation of a gas chromatography–mass spectrometry method for determination of 30 fragrance substances in cosmetic products. SSC plus.

[18] Hewala I, El-Faratre H, Emam E, Mubrouk M (2010). Development and application of a validated stability-indicating HPLC method for simultaneous determination of granisetron hydrochloride, benzyl alcohol and their main degradation products in parenteral dosage forms. Talanta.

[19] Ureta-Zañartu MS, Berríos C, González T, Fernández F, Báez D, Salazar R, Gutiérrez C (2012). Electrocatalytic oxidation of alcohols at gold electrodes in alkaline media. Int J Electrochem Sci.

[20] Ureta-Zañartu MS, González T, Fernández F, Báez D, Berríos C, Gutiérrez C (2012). Electro-oxidation of benzyl and aliphatic alcohols on polyNiTSPc- and Ni(OH)2-modified glassy-carbon and gold electrodes. Int J Electrochem Sci.

[21] Hoogvliet JC, Dijksma M, Kamp B, van Bennekom WP (2000). Electrochemical pretreatment of polycrystalline gold electrodes to produce a reproducible surface roughness for self-assembly: a study in phosphate buffer pH 7.4. Anal Chem.

[22] Wu T, Wang C, Wang X, Ma Q (2008). Simultaneous determination of 21 preservatives in cosmetics by ultra performance liquid chromatography. Int J Cosmet Sci.

[23] Cudina OA, Comor MI, Jankovic IA (2005). Simultaneous determination of bifonazole and benzyl alcohol in pharmaceutical formulations by reverse-phase HPLC. Chromatographia.

[24] Aranowska I, Wojciechowska I, Solarz N, Krutysza E (2014). Determination of preservatives in cosmetics, cleaning agents and pharmaceuticals using fast liquid chromatography. J Chromatogr Sci.

[25] Shabir GA (2007). Method development and validation of preservatives determination (benzyl alcohol, ethylene glycol monophenyl ether, methyl hydroxybenzoate, ethyl hydroxybenzoate, propyl hydroxybenzoate, and butyl hydroxybenzoate) using HPLC. J Liq Chrom Relat Tech.

[26] Furlanetto S, Orlandini S, Giannini I, Pasquini B, Pinzauti S (2010). Microemulsion electrokinetic chromatography: an application for the simultaneous determination of suspected fragrance allergens in rinse-off products. Talanta.

[27] Elkady EF, Tammam MH, Elmaaty AA (2017). HPLC-UV vs. UPLC-DAD for estimation of tinidazole, benzyl alcohol and hydrocortisone acetate simultaneously with tioconazole and its related impurities in bulk and pharmaceutical formulations. TACL.

[28] Sanchez-Prado L, Lamas JP, Alvarez-Rivera G, Lores M, Garcia-Jares C, Llompart M (2011). Determination of suspected fragrance allergens in cosmetics by matrix solid-phase dispersion gas chromatography–mass spectrometry analysis. J Chromatogr A.

