# Supplementary material for: Electrochemical sensing platform with gold nanoparticles capped by PDDA for benzyl alcohol determination
Source: Mikrochim Acta. 2023 Mar 6;190(4):115. doi: 10.1007/s00604-023-05690-6 (PMC9988818; doi:10.1007/s00604-023-05690-6)
Supplement: Supplementary file 1 — Supplementary file1 (DOCX 461 KB) [file 604_2023_5690_MOESM1_ESM.docx]

**ELECTRONIC SUPPORTING MATERIAL**

**Electrochemical sensing platform with gold-nanoparticles capped by PDDA for benzyl alcohol determination**

*Lucía Abad-Gil (https://orcid.org/0000-0002-0296-8053), M. Jesús Gismera (https://orcid.org/0000-0002-6344-0148), M. Teresa Sevilla (https://orcid.org/0000-0003-0016-9714), Jesús R. Procopio* (https://orcid.org/0000-0003-1200-8408)*

Departamento de Química Analítica y Análisis Instrumental, Facultad de Ciencias, Universidad Autónoma de Madrid. Avda. Francisco Tomás y Valiente, 7. E-28049 Madrid, Spain

* Corresponding author e-mail: jrprocopio@uam.es

***Section S1. Chemometric optimization of the photochemical synthesis of AuNP***

The irradiation time, and the concentrations of AuCl_4_^-^ and PDDA were selected as independent variables and optimized using the response surface methodology (RSM) and the central composite design (CCD). The total number of tests required for CCD is 2*^k^* + 2$\times$*k*+ *n*, being 2*^k^* the factorial design tests (where *k* is the number of factors), 2$\times$*k* the axial points, and *n* the number of replicates of the centre point. Thus, a set of 18 experiments was required for the optimization of the three variables or factors. In the CCD, 5 levels of the studied factors were evaluated: ±1, 0, ±1.682 (±α, α being 2^k/4^) for the factorial-design experiments, centre points, and axial-points, respectively [S1, S2]. Table S1 shows the experimental design for the optimization of the three variables, including the values for the factors that were selected considering the conditions used in previous works [S3-S9]. Experiments from 1 to 8 represent the factorial design tests that give information about first order effects and the interactions between the different factors [S1, S2]. The axial points are the experiments between 10 and 15, these tests give information about second order effects and are at a distance α from the design centre [S1, S2]. Experiments 9 and 16 to 18 are the centre point and its replicates tests, respectively, and give information about the experimental error [S1, S2].

**Table S1**. Central Composite Design for the optimization of three variables

| Experiment | Coded values | | | Actual values | | | I (µA) |
| --- | --- | --- | --- | --- | --- | --- | --- |
|  | x_1_ | x_2_ | x_3_ | t (min) | [AuCl_4_^-^]  (mol L^-1^) | [PDDA] (%) |  |
| 1 | 1 | -1 | -1 | 28 | 3.20×10^-4^ | 0.72 | 0 |
| 2 | 1 | -1 | 1 | 28 | 3.20×10^-4^ | 2.7 | 10.8 |
| 3 | -1 | -1 | -1 | 8 | 3.20×10^-4^ | 0.72 | 5.43 |
| 4 | -1 | -1 | 1 | 8 | 3.20×10^-4^ | 2.7 | 6.22 |
| 5 | 1 | 1 | 1 | 28 | 1.10×10^-3^ | 2.7 | 5.84 |
| 6 | 1 | 1 | -1 | 28 | 1.10×10^-3^ | 0.72 | 8.14 |
| 7 | -1 | 1 | 1 | 8 | 1.10×10^-3^ | 2.7 | 5.63 |
| 8 | -1 | 1 | -1 | 8 | 1.10×10^-3^ | 0.72 | 8.40 |
| 9 | 0 | 0 | 0 | 18 | 7.20×10^-4^ | 1.7 | 13.9 |
| 10 | 0 | +1.682 | 0 | 18 | 1.40×10^-3^ | 1.7 | 4.18 |
| 11 | 0 | 0 | +1.682 | 18 | 7.20×10^-4^ | 3.4 | 4.41 |
| 12 | 0 | -1.682 | 0 | 18 | 2.00×10^-5^ | 1.7 | 0 |
| 13 | 0 | 0 | -1.682 | 18 | 7.20×10^-4^ | 0.01 | 0 |
| 14 | +1.682 | 0 | 0 | 35 | 7.20×10^-4^ | 1.7 | 7.12 |
| 15 | -1.682 | 0 | 0 | 1 | 7.20×10^-4^ | 1.7 | 0 |
| 16 | 0 | 0 | 0 | 18 | 7.20×10^-4^ | 1.7 | 11.7 |
| 17 | 0 | 0 | 0 | 18 | 7.20×10^-4^ | 1.7 | 13.9 |
| 18 | 0 | 0 | 0 | 18 | 7.20×10^-4^ | 1.7 | 12.4 |

The different tests were randomly performed and suspensions with different characteristics depending on synthesis conditions were obtained. Table S1 also presents the anodic current of 200 µg mL^-1^ benzyl alcohol solution in 0.10 mol L^-1^ KOH obtained from cyclic voltammograms on SPCE modified with the different suspensions. In the voltammograms (Figure S1), an anodic peak at +0.017±0.003 V (*vs* Ag) and a cathodic peak at -0.107±0.006 V (*vs* Ag) due to the oxidation and reduction of benzyl alcohol, respectively, were observed


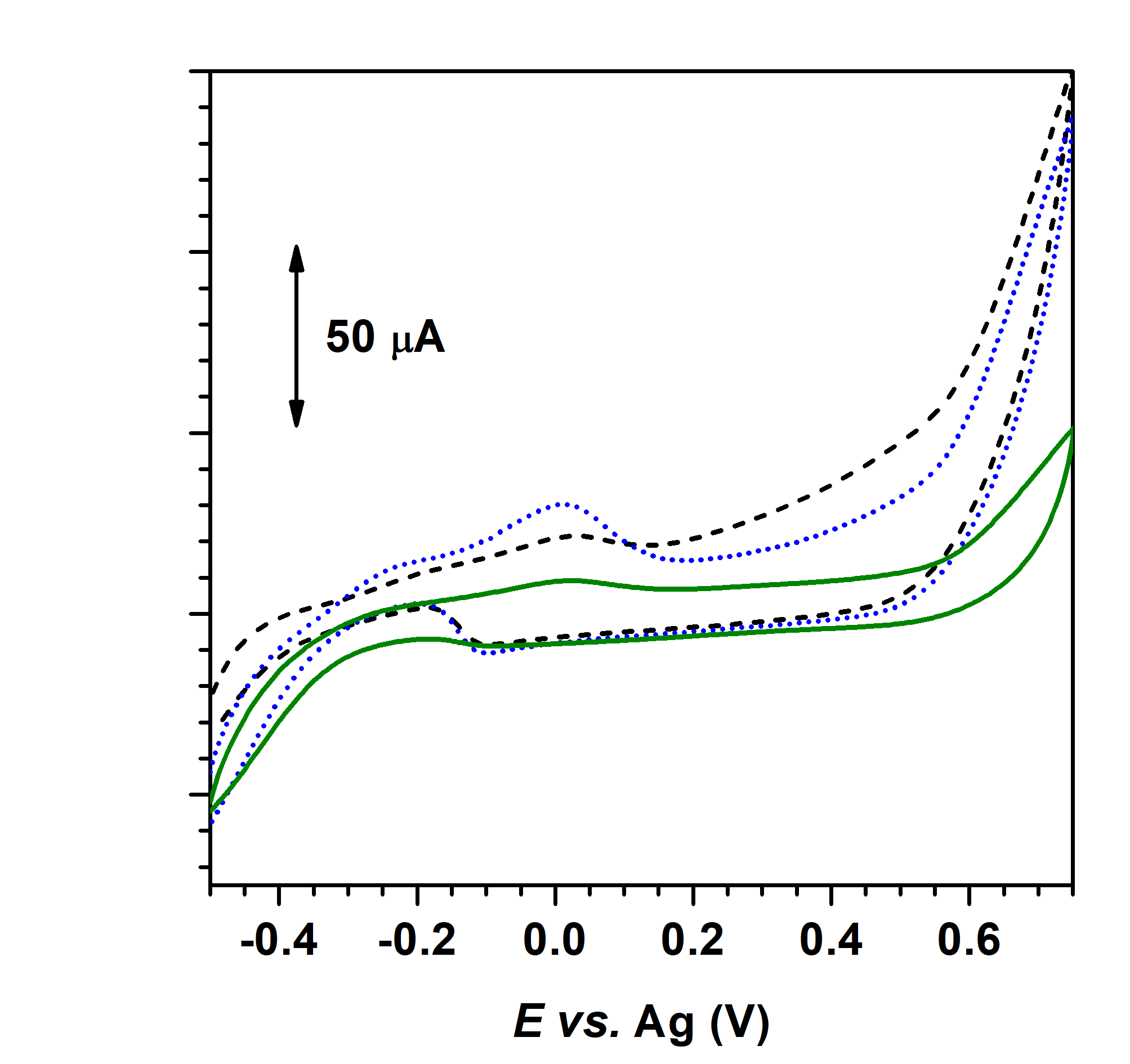


**Figure S1.** Cyclic voltammograms of 200 µg mL^-1^ benzyl alcohol in 0.10 mol L^-1^ KOH on SPCE modified with AuNP suspensions obtained in experiment 3 (**---**), experiment 10 (**―**), and experiment 18 (**···**). Scan rate: 0.100 V s^-1^.

To select the AuNP suspension with the best characteristics to develop the electrochemical sensor, the second order polynomial equation that relate the electrochemical response of benzyl alcohol with the studied factors was obtained. From the experimental results (Table S1), the following second order polynomial equation in terms of the coded factors was obtained using the least square method:

$y=12.79+0.4680x_{1}+0.5819x_{2}+1.240x_{3}-2.404x_{1}^{2}-2.922x_{2}^{2}-2.883x_{3}^{2}+0.1001x_{1}x_{2} +1.308x_{1}x_{3}-2.082x_{2}x_{3}$ ***(1)***

Where *x_1_* is the irradiation time, *x_2_* the concentration of the metal precursor, and *x_3_* the concentration of PDDA.

To evaluate the significance of the coefficients of the mathematical model ***b_j_*** (where are included the lineal (***b_i_***), the quadratic (***b_ii_***) and the interaction coefficients (***b_ij_***)) the value of the experimental *t*-student (***t_j_***) was calculated using the following equations:

$\boldsymbol{t}_{\boldsymbol{j}}\boldsymbol{=}\frac{\left| \boldsymbol{b}_{\boldsymbol{j}} \boldsymbol{coefficients} \right|}{\boldsymbol{\sigma}_{\boldsymbol{bj}}}$ ***(2)***

$\boldsymbol{\sigma}_{\boldsymbol{bj}}^{\boldsymbol{2}}\boldsymbol{=}\boldsymbol{C}_{\boldsymbol{jj}}\boldsymbol{\sigma}_{\boldsymbol{rep}}^{\boldsymbol{2}}$ ***(3)***

$\boldsymbol{\sigma}_{\boldsymbol{rep}}^{\boldsymbol{2}}\boldsymbol{=}\frac{\sum_{\boldsymbol{i}\boldsymbol{=}\boldsymbol{1}}^{\boldsymbol{n}_{\boldsymbol{0}}} \left( \boldsymbol{y}_{\boldsymbol{i}}\boldsymbol{-}{\bar{\boldsymbol{y}}}_{\boldsymbol{0}} \right)^{\boldsymbol{2}}}{\boldsymbol{n}_{\boldsymbol{0}}\boldsymbol{-}\boldsymbol{1}}$ ***(4)***

Where ***σ_bj_^2^*** is the variance of the coefficients, ***C_jj_*** are the diagonal terms of [X^T^·X]^-1^matrix, ***σ_rep_^2^*** is the variance of the centre point replicates, ***y_i_*** is the response of the centre points, ***ӯ_0_*** is the mean response in the centre point, and ***n_0_*** is the number of replicates of the centre-point.

According to the Student’s t-test (Table S2), the ***t_j_*** values of the coefficients ***b_1_*** (0.4680) corresponding to the individual effect of the irradiation time, ***b_2_*** (0.5819) corresponding to the individual effect of concentration of the metal precursor, and ***b_12_*** (0.1001) corresponding to the interaction between the irradiation time and concentration of metal precursor, are lower than the ***t*-critical** (3.18, at 95% confidence level and 3 degrees of freedom). Thus, these coefficients are considered not significant and excluded from the polynomial equation.

**Table S2.** Student’s t-test of the mathematical model

|  | Coefficient | *Cjj* | *σ_bj_* | *t_j_* |
| --- | --- | --- | --- | --- |
| *b_0_* | 12.79 | 0.177 | 0.468 | 27.3 |
| *b_1_* | 0.4680 | 0.0732 | 0.301 | 1.58 |
| *b_2_* | 0.5819 | 0.0732 | 0.301 | 1.94 |
| *b_3_* | 1.240 | 0.0732 | 0.301 | 4.13 |
| *b_1_^2^* | -2.404 | 0.0500 | 0.249 | 9.66 |
| *b_2_^2^* | -2.922 | 0.0500 | 0.249 | 11.7 |
| *b_3_^2^* | -2.883 | 0.0500 | 0.249 | 11.6 |
| *b_12_* | 0.1001 | 0.125 | 0.393 | 0.255 |
| *b_13_* | 1.308 | 0.125 | 0.393 | 3.33 |
| *b_23_* | -2.082 | 0.125 | 0.393 | 5.30 |

***t*** *critical (95% confidence level)* = 3.18

Therefore, the equation that describes the response of the system is the following:

$y=12.79+1.240x_{3}-2.404x_{1}^{2}-2.922x_{2}^{2}-2.883x_{3}^{2} +1.308x_{1}x_{3}-2.082x_{2}x_{3}$ ***(5)***

The coefficient of determination (*R^2^*) was also calculated to study the correlation between the experimental data and those obtained using the mathematical model (Equation 5). The *R^2^* value of the obtained equation is 0.8595 (at 95%-confidence level), so an accurate response can be obtained with the proposed mathematical model.

The reliability of the method was also studied using the Fisher test (equations 6 and 7).

$\boldsymbol{F}\boldsymbol{=}\frac{\boldsymbol{\sigma}_{\boldsymbol{res}}^{\boldsymbol{2}}}{\boldsymbol{\sigma}_{\boldsymbol{rep}}^{\boldsymbol{2}}}$ ***(6)***

$\boldsymbol{\sigma}_{\boldsymbol{res}}^{\boldsymbol{2}}\boldsymbol{=}\frac{\sum_{\boldsymbol{i}\boldsymbol{=}\boldsymbol{1}}^{\boldsymbol{N}} \left( \boldsymbol{y}_{\boldsymbol{i}}\boldsymbol{-}{\hat{\boldsymbol{y}}}_{\boldsymbol{i}} \right)^{\boldsymbol{2}}}{\boldsymbol{N}\boldsymbol{-}\boldsymbol{l}}$ ***(7)***

Being ***σ_res_^2^*** the residual variance, ***N*** the number of experiments, ***l*** the number of coefficients in the polynomial equation, ***y_i_*** the response observed in each experiment, and ***ŷ_i_*** the estimated response by the mathematical model.

As can be seen in Table S3 the ***F*** value was lower than ***F* critical** (at 95% confidence level, and 11 and 3 degrees of freedom for the numerator and denominator, respectively). This result suggests that there are no significant differences between the variances and thus the mathematical model is adequate to predict the responses.

**Table S3.** Fisher test of the mathematical model

| Residual variance *(σ_res_^2^)* | 9.70 |
| --- | --- |
| Variance of the replicates *(σ_rep_^2^)* | 1.24 |
| Number of experiments *(N)* | 18 |
| Number of coefficients *(l)* | 7 |
| *F* value | 7.84 |
| *F* critical value | 8.76 |

From the obtained mathematical model (equation 5), the response surfaces were constructed using the *Matlab* software (Figure S2).


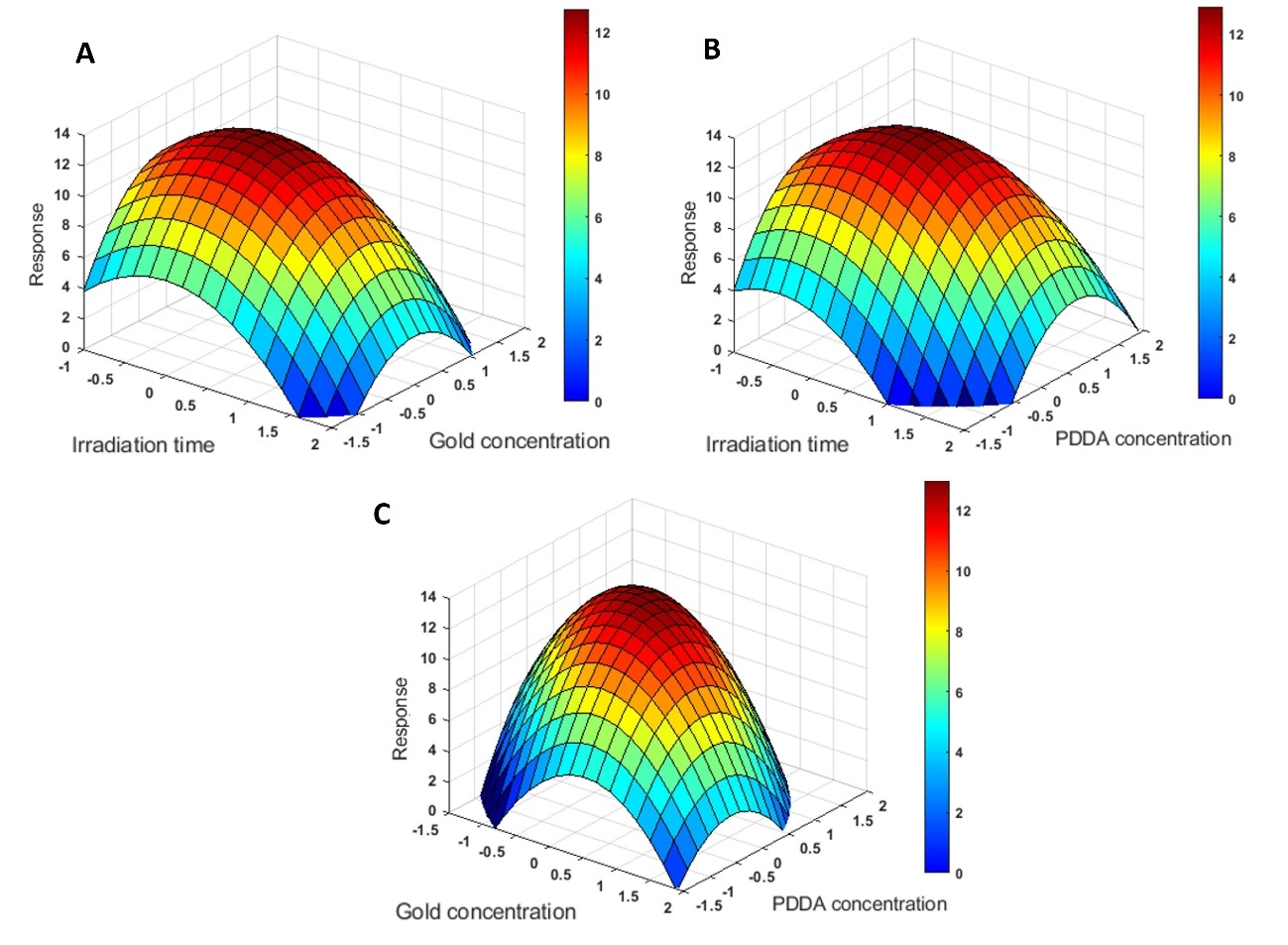


**Figure S2.** Response surfaces obtained in the optimization of the photochemical synthesis of AuNP. (A) Effect of the irradiation time and AuCl_4_^-^ concentration, maintaining PDDA concentration constant (1.7%, *x_3_*=0). (B) Effect of the irradiation time and PDDA concentration, maintaining AuCl_4_^-^ concentration constant (7.20 $\times$10^-4^ mol L^-1^, *x_2_*=0). (C) Effect of AuCl_4_^-^ and PDDA concentration, maintaining the irradiation time constant (18 min, *x_1_*=0).

**References:**

1. Bezerra M.A., Santelli R.E., Oliveira E.P., Villar L.S., Escaleira L.A. (2008) Response surface methodology (RSM) as a tool for optimization in analytical chemistry. Talanta, 76: 965-977. DOI:10.1016/j.talanta.2008.05.019.
2. Aslan. (2008) Application of response surface methodology and central composite rotatable design for modelling and optimization of a multi-gravity separator for chromite concentration. Powder Technol., 185: 80-86. DOI:10.1016/j.powtec.2007.10.002
3. Jara N., Milán N.S., Rahman A., Mouheb L., Boffito D.C., Jeffryes C., Dahoumane S.A. (2021) Photochemical synthesis of gold and silver nanoparticles—A Review. Molecules, 26: 4585. DOI: 10.3390/molecules26154585.
4. Abad-Gil L., Gismera M.J., Sevilla M.T., Procopio J.R. (2020) Methylisothiazolinone response on disposable electrochemical platforms modified with carbon, nickel or gold-based nanomaterials. Microchim. Acta, 187: 199. DOI: 10.1007/s00604-020-4157-3.
5. Mevold A.H.H., Hsu W.W, Hardiansyah A., Huang L.Y., Yang M.C., Liu T.Y., Chan T.Y., Wang K.S., Su Y.A., Jeng R.J., Wang J.K., Wang Y.L. (2015) Fabrication of gold nanoparticles/ graphene-PDDA nanohybrids for biodetection by SERS nanotechnology. Nanoscale Res. Lett., 10:397. DOI 10.1186/s11671-015-1101-2
6. Xiao J., Zhang T., Li R., Meng Y., Wen W. (2012) Surface-Enhanced Raman Scattering for quantitative analysis of perchlorate using poly(diallyldimethylammonium chloride) capped gold nanoparticles. Appl. Spectrosc., 66: 1027-1033. DOI: 10.1366/12-06645
7. Sau T. K., Pal A., Jana N.R., Wang Z.L., Pal T. (2001) Size controlled synthesis of gold nanoparticles using photochemically prepared seed particles. J. Nanopart. Res., 3: 257–261. DOI: 10.1023/A:1017567225071
8. Huang W.C., Chen Y.C. (2008) Photochemical synthesis of polygonal gold nanoparticles. J. Nanopart. Res., 10:697–702. DOI: 10.1007/s11051-007-9293-8
9. Sun X., Dong S., Wang E. (2004) One-step synthesis and characterization of polyelectrolyte-protected gold nanoparticles through a thermal process. Polymer, 45: 2181–2184. DOI: 10.1016/j.polymer.2004.01.010

**
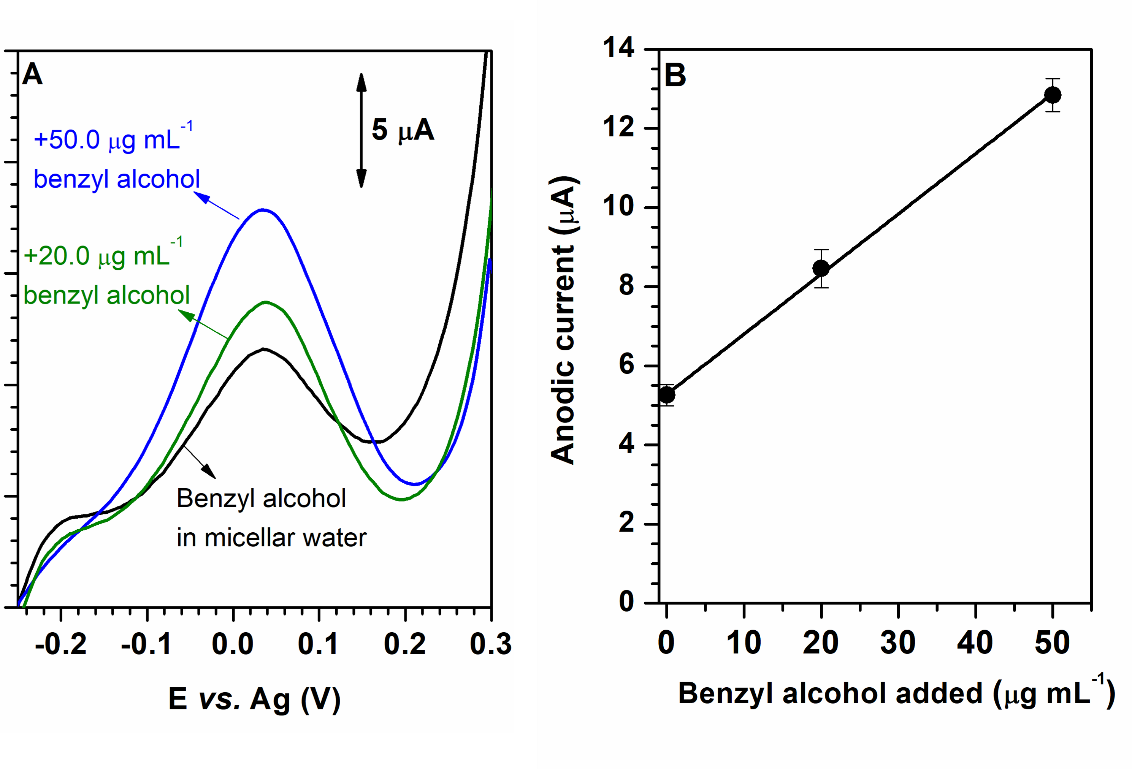
**

**Figure S3** Determination of benzyl alcohol in a micellar water by LSV in 0.10 mol L^-1^ KOH on the AuNP@PDDA/SPCE sensor by standard addition method (A) Linear sweep voltammograms and (B) Calibration plot.
